# Pharmaceutical distribution remuneration in Europe

**DOI:** 10.1186/2052-3211-8-S1-P23

**Published:** 2015-10-05

**Authors:** Sabine Vogler, Lena Lepuschütz, Peter Schneider

**Affiliations:** 1 WHO Collaborating Centre for Pharmaceutical Pricing and Reimbursement Policies, Health Economics Department, Gesundheit Österreich GmbH (Austrian Public Health Institute), Vienna, 1010, Austria

## Background

Medicine prices are considerably influenced by the maximum remuneration allowed to wholesalers and pharmacies. Remuneration of pharmaceutical distribution has to strike a balance between appropriate compensation for supply chain management and further services, while ensuring sustainability of public funding.

## Objectives

The study aims to survey and compare remuneration policies for wholesale and community pharmacies for 30 European countries (all 28 EU Member States, Norway and Switzerland).

## Methods

We performed a primary data collection with public authorities, wholesale and pharmacy associations as well as Internet and literature research. The analysis focuses on the reimbursement segment (i.e. full or partial cost coverage of public payers) of the out-patient sector, using data for the first quarter of 2015.

## Results

The data show that in Cyprus, Denmark, Finland, the Netherlands, Norway, Sweden and the UK wholesale remuneration is negotiated between manufacturers and wholesalers. In the remaining countries, a maximum allowed wholesale remuneration is regulated on a legal basis, at least for reimbursable medicines. In these countries, wholesale remuneration generally depends on the price of the medicine. Regressive schemes have become more common than linear margins/mark-ups.

In 28 of the 30 countries surveyed, pharmacy remuneration is regulated or agreed upon between the pharmacy sector and payers at least for reimbursable medicines, whereas in Cyprus and Malta pharmacy remuneration is not an issue as pharmacies are state-owned and directly belong to public sector. In 16 of these 28 countries, remuneration depends solely on the price of the dispensed medicine, and is usually a regressive scheme. A purely performance-based fee-for-service remuneration is in place in five countries (Croatia, Ireland, the Netherlands, Slovenia and the UK). Pharmacy remuneration that is both price-based and performance-based exists in seven countries (Belgium, Denmark, Finland, France, Germany, Norway and Switzerland).

## Conclusions

Results show that policies implemented in European countries to compensate pharmaceutical distribution actors are similar. Frequently, remuneration is dependent on the price; this might incentivize the supply and/or dispensing of higher-priced medicines. In the community pharmacy sector, new pharmacy remuneration models have been put in place which are disconnected to the medicine price and support a wider role of pharmacists as health care provider. Changes in wholesale and pharmacy remuneration in recent years were done for cost-containment reasons in response to the crisis as well as for designing new remuneration models that are better aligned to the current challenges of supply management and dispensing of medicines.

